# Dark Matter Carried by *Sinorhizobium meliloti* phiLM21-like Prophages

**DOI:** 10.3390/ijms26178704

**Published:** 2025-09-06

**Authors:** Maria E. Vladimirova, Marina L. Roumiantseva, Alla S. Saksaganskaia, Alexandra P. Kozlova, Victoria S. Muntyan, Sergey P. Gaponov

**Affiliations:** 1 Laboratory of Genetics and Selection of Microorganisms, Federal State Budget Scientific Institution All-Russia Research Institute for Agricultural Microbiology (FSBSI ARRIAM), 196608 Saint Petersburg, Russia; mariiacherkasova@arriam.ru (M.E.V.); allasaksaganskaya@arriam.ru (A.S.S.); a.kozlova@arriam.ru (A.P.K.); vucovar@arriam.ru (V.S.M.); 2 Novikov Labs, 420033 Kazan, Russia; bioisergey@gmail.com

**Keywords:** temperate *Sinorhizobium* phage phiLM21, lysogenic/lytic cycle, non-pathogenic bacteria, intact *Sinorhizobium meliloti* prophages, nucleotide and amino acid sequences, phylogenetic analysis, integrases, phage repressors (regulators), anti-phage defense systems, dark matter of genetic origin

## Abstract

A comprehensive comparative analysis was conducted on the nucleotide and amino acid sequences of intact phiLM21-like prophages (phiLM21-LPhs), which currently represent the most prevalent prophages in *Sinorhizobium meliloti*—a symbiotic partner of *Fabaceae* plants. Remarkably, the nucleotide sequences of 25 phiLM21-LPhs, identified across 36 geographically dispersed *S. meliloti* strains, covered no more than 34% of the phiLM21 phage genome. All prophages were integrated into specific isoacceptor tRNA genes and carried a tyrosine-type integrase gene; however, this integration did not exhibit features of tRNA-dependent lysogeny. Only one-fifth of phiLM21-LPhs encoded the minimal set of regulators for lysogenic/lytic cycle transitions, while the remainder contained either uncharacterized regulatory elements or appeared to be undergoing genomic “anchoring” within the host bacterium. The phiLM21-LPhs harbored open reading frames (ORFs) of diverse origins (phage-derived, bacterial, and unknown), yet over half of these ORFs had undeterminable functions, representing genetic “dark matter”. The observed diversification of intact phiLM21-like prophages likely stems from recombination events involving both virulent/temperate phages and phylogenetically remote bacterial taxa. The evolutionary and biological significance of the substantial genetic “dark matter” within these prophages in soil saprophytic bacteria remains an unresolved question.

## 1. Introduction

Bacteriophages, as the most numerous group of viruses, are widely found in all ecosystems [1,2,3,4]. However, soil phages are far less studied than phages from other ecosystems [5,6,7]. Viral particles reach their highest density in the root zone and directly in the rhizosphere of plant roots [8,9].

The infectious activity of phages can exceed 10^23^ bacterial cells per second [10]. As is known, phages lyse bacteria but can also integrate into the host bacterium’s genome via *attB* attachment sites (site-specific integration), which are found on both chromosomes and plasmid replicons (prophage; [11]). This property of phages has contributed to the active development of nature-like technologies aimed at delivering target genes to specific genomic locations using phage vectors [12,13].

Currently, most information about bacterial prophages—including their structure, prevalence, and functional significance—has been accumulated through studies of pathogenic or conditionally pathogenic bacterial strains, such as *Escherichia coli*, *Bacillus* spp., *Pseudomonas* spp., *Streptococcus pyogenes*, *Salmonella enterica*, and *Staphylococcus aureus*, primarily driven by medical interests. For example, it has been shown that prophages can be present in significant numbers in bacterial genomes, accounting for up to 10–20% of their genome [14,15,16]. The genome of *E. coli* can contain up to 18 prophage sequences of varying integrity, while strains of *Desulfovibrio vulgaris* may carry up to 6 sequences [17,18]. Our research has revealed that in the genomes of non-pathogenic nodule bacteria, *Sinorhizobium meliloti*, the number of prophages per genome can reach 30 [11,19].

By integrating into the host bacterium’s genome, temperate (lysogenic) phages can persist for extended periods as prophages, replicating alongside the bacterial genome and thus being inherited vertically. The involvement of prophages in horizontal gene transfer depends on their ability to excise, which is often triggered by adverse environmental factors affecting the host cell (prophage induction). Prophage induction leads to lytic development, starting with prophage excision from the bacterial genome and culminating in the formation of viral particles and cell lysis. During excision, the prophage can carry not only its own genes encoding “typical” phage proteins but also fragments of the host bacterium’s DNA, such as metabolic genes [20,21].

Moreover, prophage sequences actively participate in intracellular recombination processes [22,23,24]. This explains why phages often contain sequences from host bacteria as well as gene clusters from different phage lineages [22,23,24]. Such transfer facilitates the emergence of new phage genetic variants while also expanding the genetic potential of bacteria, enabling them to acquire new traits (phage conversion). For instance, prophages may carry genes responsible for antibiotic resistance, virulence, or the synthesis of various metabolites, thereby influencing the host bacterium’s resistance to adverse conditions and its overall viability [4,25,26].

Phage genomes are mosaic, meaning they consist of gene clusters from phages of different genetic lineages [22,23,24]. As a result, phages rapidly lose their authenticity. A striking example is the temperate rhizobiophage 16-3, isolated in the 1960s from the strain *Rhizobium* (*Sinorhizobium*) *meliloti* 41 in Hungary [19,27]. The genome of this phage serves as a model for genetic studies of homoimmune infection [28].

Today, sequences similar to those of phage 16-3 occur at a frequency of 0.09 within prophages of modern *S. meliloti* strains or other members of the *Rhizobiaceae* family, as well as in aquatic viromes from Ireland (NCBI database, 09.2022 [19]). This suggests that the temperate phage 16-3 was once widely distributed in the genomes of root-nodulated bacteria in the mid-20th century. However, its sequences are now virtually absent, possibly due to active recombination processes and the efficiency of bacterial host anti-phage defense systems, including restriction–modification systems.

Nevertheless, 10 extended sequences (ranging from 32 kb to 104.2 kb) related to phage 16-3, exhibiting low homology, have been identified in the genomes of various bacterial strains, including *Mesorhizobium* spp., *Rhizobium leguminosarum*, and an *S. meliloti* strain isolated from saline soils in the Aral Sea region [19]. Analysis of these phage 16-3-related sequences revealed that 81% of the identified open reading frames (ORFs) were either genes from diverse phages (89 phages infecting 45 bacterial species) or genes encoding hypothetical proteins. The latter is of particular interest, as such “dark” genetic information may, through genetic rearrangements (deletion/integration/recombination), acquire functional significance and unpredictably affect the host bacterium’s metabolism [29].

Currently, the Virus–Host DB contains 17 sinorhizobiophage genomes (phiLM21, 16-3, StopSmel, Aussie, PBC5, HMSP1-Susan, NV1.1.1, phiM6, AP-16-3, AP-J-162, phiM5, ort11, phiM9, phiM19, phiM7, phiM12, and phiN3; [30], accessed 4 April 2025). Among them is *Sinorhizobium* phage phiLM21, induced from the strain *Sinorhizobium* sp. LM21 (BioSample ID: SAMN06765771), isolated in 2014 from soils of a copper mine in Poland. Phage phiLM21 was characterized as having a siphovirus morphology (icosahedral head, flexible non-contractile tail, double-stranded DNA) [31]. According to the new phage classification [32], phiLM21 belongs to the unclassified *Caudoviricetes* (Virus–Host DB; [30]). Our study has shown that intact prophages like phiLM21 are widespread in the genomes of modern *S. meliloti* strains isolated from geographically remote regions, far from both each other and the original isolation site of phiLM21 [11].

The goal of our study was to conduct a systematic analysis of intact phiLM21-like prophages prevalent in the genomes of modern *Sinorhizobium meliloti* strains (symbionts of economically valuable leguminous plants). This approach provides new insights into the diversification of phage-derived sequences in the host bacterial genome and assesses the significance of phage conversion by analyzing the functional significance of “captured” foreign genetic material.

## 2. Results

In this study, we focused on assessing the diversification/“conservation” of nucleotide and amino acid sequences of intact prophages that are related to the temperate phage phiLM21, which appears to be an active participant in phage–microbe interactions within modern microcenosis.

Analysis of 36 *S. meliloti* genomes (see Section 4: Materials and Methods) revealed that 13 genomes contained a single sequence similar to phiLM21 (GenBank accession number NC_029046), while 6 genomes harbored two such sequences. Consequently, the further analysis was conducted on 25 intact phiLM21-like sequences (hereafter referred to as phiLM21-like prophages or phiLM21-LPhs; Table 1). According to PHASTEST classification, ‘intact’ phiLM21-LPhs are similar to phiLM21 in size and the number of phage genes/proteins, and they contain a set of ‘cornerstone’ genes. The phage origin proteins encoded in phiLM21-LPhs are predominantly similar to those of phiLM21 (Table 1).

All identified phiLM21-LPhs were integrated into specific isoacceptor tRNA genes. It has been shown that the host bacterial tRNA genes (integration sites) were reconstructed during the integration of phiLM21-LPhs. The most frequently involved was the tRNA-Lys(CUU) gene (frequency 0.52), followed by the tRNA-Ser(GCU) gene (frequency 0.28), and, less commonly, the tRNA-Leu(UAA) gene (frequency 0.16). In a single case, phiLM21-LPh was integrated into the same tRNA gene as phage phiLM21, specifically the tRNA-Pro(GGG) gene (frequency 0.04) (Table 1). These tRNA genes were numbered 039, 016, 031, and 017, corresponding to their positions on the chromosome relative to *oriC*, based on the analysis of the structural organization of the chromosome in bacteria of the species *S. meliloti* [11]. Each of the studied phiLM21-LPhs was named based on the strain in which it was identified and the order number of the tRNA gene into which the prophage was integrated (Table 1).

### 2.1. Analysis of Nucleotide Sequences

A comparative analysis of the characteristics of nucleotide sequences of phiLM21-LPhs with phiLM21, as well as with the complete genome sequences of 16 phages infecting *Sinorhizobium* spp. (see Section 4: Materials and Methods), revealed that their length ranged from 50,261 to 55,743 base pairs (average length 53.0 ± 0.25 kb). However, on average, this was no more than 10% longer or, at most, 1% shorter relative to the phiLM21 sequence (50,827 bp). On average, each phiLM21-LPh contained 75 ± 1 ORFs (genes) (Table 1). In 15 phiLM21-LPhs, a single tRNA gene was identified, represented by 10 tRNA-Met(CAU), 4 tRNA-fMet(CAU), and, in one case, a tRNA-Val(CAC). Only phiM162-016 contained two genes, specifically tRNA-Met(CAU) and tRNA-fMet(CAU). The GC content in phiLM21-LPhs varied from 59.2% to 60.8% (mean 60.0 ± 0.1%), comparable to that of phiLM21 (60.6%).

**Figure 1:**
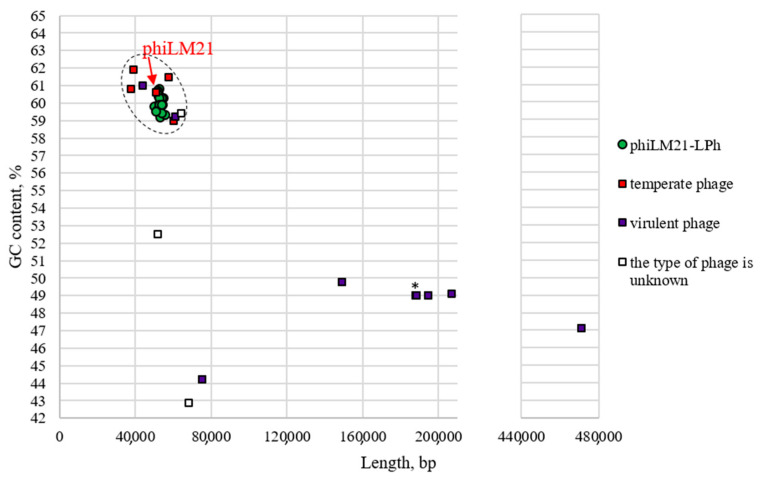
The scatter plot illustrates the distribution of 25 phiLM21-like prophages, the phiLM21 phage itself, and 16 phages that infect *S. meliloti*. On the x-axis, genome lengths (in base pairs) are plotted, while the y-axis represents GC content (%) for phiLM21-LPhs, phiLM21, and the 16 *S. meliloti*-infecting phages (see Section 4: Materials and Methods). Values ranging from 200 to 440 kb on the x-axis are not shown, as phages infecting *S. meliloti* with such genome sizes are currently unknown. A red arrow indicates the position of the temperate phage phiLM21 on the plot. The dotted line highlights a cloud of dots formed by currently known temperate phages (phiLM21, StopSmel, Aussie, 16-3, PBC5). Additionally, an asterisk (*) marks a point on the diagram corresponding to two phages, phiM19 and phiM7, which have similar genome lengths and GC content.

Scatter plot analysis revealed that the genome length and GC content of phiLM21-LPhs (53.0 ± 0.25 kb, 60.0 ± 0.1%) are similar to its temperate progenitor, phiLM21 (50.8 kb, 60.6%) (Figure 1). The phiLM21-LPhs variants co-clustered with other temperate *Sinorhizobium* phages (e.g., StopSmel, Aussie, 16-3, PBC5) and were comparable to two virulent phages (AP-16-3, phiM5) and to NV1.1.1. In contrast, phiLM21-LPhs were significantly different from the seven other virulent phages studied, which had distinctly larger genomes and lower GC content (210.4 ± 46.6 kb, 48.2 ± 0.7%) (Figure 1).

Thus, all identified phiLM21-LPhs are more similar to phiLM21 in their length and nucleotide composition than to other phages infecting *S. meliloti*. The obtained result confirms data previously reported by researchers of the phiLM21 phage, indicating that its sequence had limited similarity to other known phage genomes [31].

The GC content of phiLM21-LPhs, phiLM21, and four temperate rhizobiophages (StopSmel, Aussie, 16-3, PBC5) was found to be close to that of the host bacterium (the average GC content of the genome of the reference strain *S. meliloti* 1021 is 62%), compared to the GC content of virulent rhizobiophages. Interestingly, the GC content values of phiLM21-LPhs and phiLM21 were closer to that of megaplasmid pSymA (60.5%) than to similar values for the chromosome or megaplasmid pSymB (both with GC content of 62.5%). For comparison, the GC content for temperate phages was as follows: for *Rhizobium* phage 16-3, it was 59%; for Aussie, 61.9%; and for PBC5, 61.5%, which was closer to the GC content of the chromosome/pSymB.

**Figure 2:**
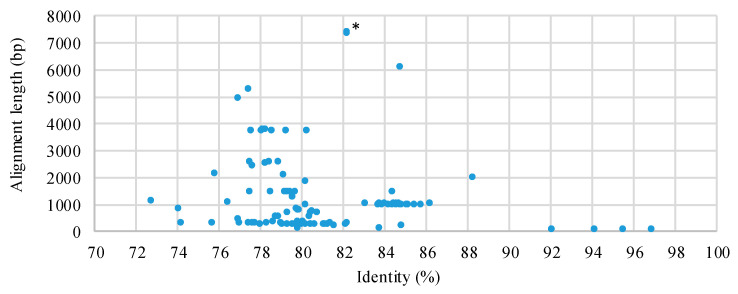
Pairwise alignment of the 25 phiLM21-LPh sequences across phiLM21. * The location of the two sequences (7347 bp and 7371 bp; I = 82.2%) in the diagram is the same.

An analysis of the nucleotide sequence similarity between phiLM21-LPhs and phiLM21 was performed. For this, the nucleotide sequence of each of the 25 phiLM21-LPhs was aligned with the phiLM21 genome sequence using BLASTn (I > 70%). As a result, each phiLM21-LPh sequence contained between 2 and 13 fragments homologous to phiLM21. The total number of fragments across the 25 phiLM21-LPhs was 116, with sizes ranging from 63 to 7371 bp (I = 72.7–96.9%) (Figure 2). A large proportion consisted of fragments smaller than 1 kb (65%), with twice as many fragments up to 4.9 kb in size (31%), and only five sequences had sizes greater than 4.9 kb (Figure 2). Detailed BLASTn alignment results are presented in Table S2.

**Figure 3:**
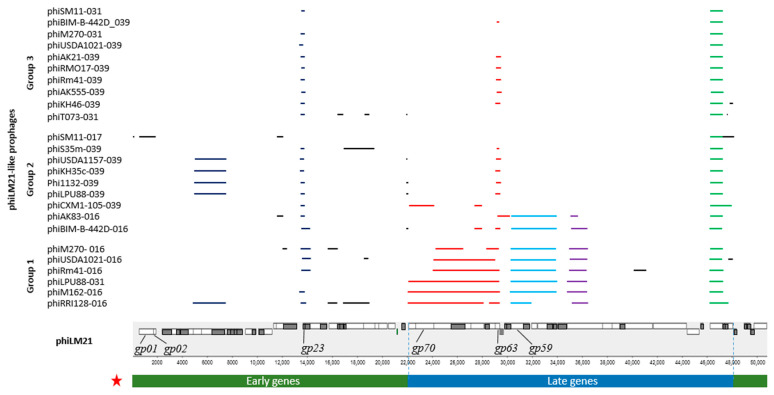
Sequences of phiLM21-LPhs similar to phiLM21. The groups and the list of corresponding phiLM21-LPhs are shown on the left (see text). Colored lines indicate sequences identified in phiLM21-LPhs, with identity to phiLM21 exceeding 70%. Red star symbol (★). For the phiLM21 phage (50,827 bp; NCBI RefSeq: NC_029046.1 from 1 June 2025), regions of phage genes associated with early and late expression were predicted based on analysis of gene-encoded protein products. The early gene expression region in phiLM21 is tentatively starting from gene *AWJ26_gp01* (*gp01*), which encodes an integrase, and *AWJ26_gp02* (*gp02*), encoding an excisionase; it includes gene *AWJ26_gp23* (*gp23*) encoding a hypothetical protein, and ends with a region encoding a transcription antiterminator and HNH endonuclease (positions 1–21,013 bp); this region also contains a non-homologous end-joining DNA ligase involved in DNA repair (positions 49,750–50,742 bp). The late gene expression region of phiLM21 is presumed to start with gene *AWJ26_gp70* (*gp70*), encoding the terminase large subunit (involved in DNA packaging into the capsid); it includes gene *AWJ26_gp63* (*gp63*), encoding head closure protein Hc1; gene *AWJ26_gp59* (*gp59*), encoding head morphogenesis; and ends with a gene encoding a membrane protein (positions 22,088–48,048 bp). Colored rectangles indicate genes with predicted functions (total of 37 genes); gray rectangles represent genes encoding hypothetical proteins (total of 35 genes); and the green rectangle indicates the tRNA-Met gene. Detailed BLASTn alignment results are presented in Table S2.

All 25 phiLM21-LPhs were divided into 3 groups according to the nucleotide similarity and coverage of the phiLM21 genome (Figure 3). Only Group 1 prophages exhibited the highest degree of similarity to phiLM21 (Figure 3; Table 2), with 4 to 10 extended genomic fragments identified in each of these prophages that were homologous to regions of the phiLM21 genome containing genes with early but predominantly late expression patterns (I = 78.6–80.7%, Cov = 22.1–33.8% of the phiLM21 genome). A prominent example is the prophage phiRRI128-016, which contained genes from both early and late expression modules, including genes involved in replication and recombination (DnaB-like replicative helicase, primosomal protein, Erf-like ssDNA annealing protein, DNA methyltransferase), excision (exonuclease), virion assembly (portal protein, head decoration protein, structural protein, head-tail adaptor Ad1, head closure protein Hc1, head morphogenesis protein, tail length tape measure protein, tail proteins), DNA packaging (small and large terminase subunits), and host lysis and particle release (chitinase), as detailed in Table S3.

Prophages in Group 1 contained sequences similar to just 31 phiLM21 genes. Five phiLM21-LPhs shared 17–18 of these genes, whereas phiRRI128-016 contained 22. All six intact prophages consistently shared seven genes from the phiLM21 late-expression module, including those for structural proteins (portal, head-tail adaptor Ad1, head morphogenesis, tail tape measure), a chitinase, and two hypothetical proteins (Table S3).

Groups 2 and 3 included 9 and 10 phiLM21-LPhs, respectively. In the case of Group 2, these were sequences that showed similarity to different parts of the phiLM21 phage genome, but, overall, they covered no more than 7.3–15% of the phiLM21 phage genome (Figure 3). For Group 3, short sequences corresponding to the same regions of the phiLM21 genome were identified within the phiLM21-LPhs, covering only 2–4% of the phage genome (Figure 3). The regions identified within phiLM21-LPhs from Groups 2 and 3 mainly contained genes from the early expression group of the phiLM21 phage. Significantly less frequently, they contained individual blocks of genes encoding proteins involved in the assembly of phage particles, packaging of phage DNA into them, and release from the host cell—that is, genes of late expression, which are expressed after phage induction. Genes involved in primary attachment to the host cell (tail fiber protein) and in terminating tail capsid assembly (tail terminator), which belong to the late expression gene group, were identified only in prophages of Group 1 and two prophages of Group 2 (phiAK83-016 and phiBIM-B-442D-016) (Table S3, Figure 3). The sequence encoding chitinase (I = 80.2–86.1%), which belongs to the late expression gene group of phages, was found in all phiLM21-LPhs (Figure 3).

Thus, the analysis of nucleotide sequences of intact phiLM21-LPhs showed that sequences with the highest similarity to the phiLM21 phage genome (Cov ≤ 34%; I ≤ 80%) occurred at a frequency of no more than 0.24. These related prophage regions harbored genes homologous to those in the early, and primarily the late, expression modules of phiLM21. Nucleotide sequence analysis revealed that the intact phiLM21-LPhs have diverged significantly from the original phiLM21 phage. Furthermore, even prophages residing within the same bacterial genome differed from one another, as they belonged to the different groups defined above.

### 2.2. Phylogenetic Analysis of phiLM21-LPhs

In all 25 phiLM21-LPhs, an ORF corresponding to the gene encoding the terminase large subunit (hereafter *tls*) was identified, which is widely used to assess the phylogenetic relatedness of studied phages [33]. The sequences of the *tls* gene from these prophages, as well as the sequence of the corresponding gene AWJ26_gp70 of phiLM21 and the sequence similar to the *tls* gene found in each of the 90 phages infecting rhizobia of the genera *Bradyrhizobium*, *Mesorhizobium*, *Rhizobium*, and *Sinorhizobium*, were used for phylogenetic analysis (see Section 4: Materials and Methods and Table S4).

The amino acid sequence (hereafter aa-*tls*) similar to that of phiLM21 was detected in only four cases: in three Group 1 phiLM21-LPhs (phiRRI128-016, phiM162-016, phiLPU88-031) and in phiCXM1-105-039 from Group 2 (Table S5). In 13 prophages, aa-*tls* was similar to sequences from *Streptomyces* phage mu1/6 NC_007967 (Table S6A). In six cases, the similarity was with amino acid sequences of terminases from *Edwardsiella* phage GF-2^NC_026611^ (phiSM11-017, phiT073-031), *Shigella* phage Sf14^NC_042075^ (phiKH35c-039, phiS35m-039), as well as *Pseudomonas* phage PA10^NC_041903^ (phiRM41-016), and *Pseudomonas* phage vB_PaeM_C2_10_Ab02^NC_042113^ (phiUSDA1021-016) (Table S6A). Two cases were identified where the *tls* gene was inactivated: in one case, the *tls* gene was a site of transposase IS1480 integration (phiAK21-039), and in the other, the sequence similar to *tls* was a pseudogene (phiM270-016).

**Figure 4:**
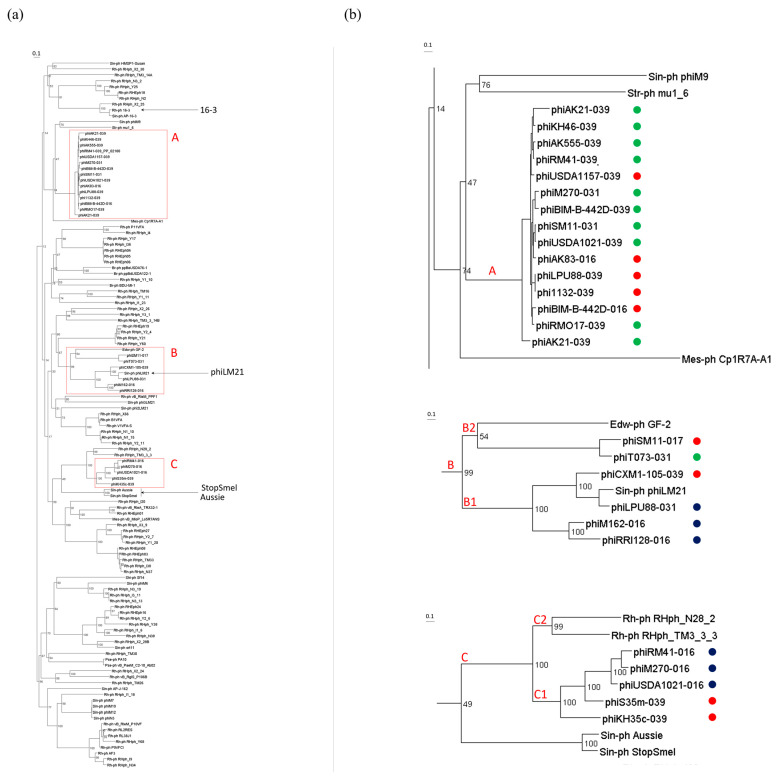
Phylogenetic tree of *tls* genes of phiLM21-LPhs and phages infecting rhizobia. (**a**) *tls* genes of the 25 phiLM21-LPhs and 90 rhizobiophages (Table S4) were used for phylogenetic analysis; (**b**) the enlarged images of the clusters A, B, and C are presented; colored balls are phiLM21-LPhs groups, blue—Group 1, red—Group 2, green—Group 3, based on nucleotide similarity with phage PhiLM21 (see Figure 3); Sin-ph—*Sinorhizobium* phage, Rh-ph—*Rhizobium* phage, Br-ph—*Bradyrhizobium* phage, Mes-ph—*Mesorhizobium* phage, Edw-ph—*Edwardsiella* phage, Str-ph—*Streptomyces* phage, Pse-ph—*Pseudomonas* phage. Phylogenetic tree (**a**) in Newick format is in the Supplementary Materials.

The phylogenetic tree shows three remote groups, clusters A, B, and C, which include all nucleotide sequences of *tls* identified within phiLM21-LPhs (Figure 4a). Cluster A was the most representative, comprising 15 *tls* sequences (bootstrap 100). However, the corresponding phiLM21-LPhs belonged to different groups (Groups 2 and 3) and were integrated into different tRNA genes (Figure 4b). The closest phylogenetic relatives of this cluster, based on cladistic analysis of the terminase sequence, were the *tls* sequences of the virulent *Sinorhizobium* phage phiM9, two *Streptomyces* phages mu1/6, and *Mesorhizobium* phage Cp1R7A-A1, infecting bacteria of the respective genera *Streptomyces* (*Actinomycetes*) and *Mesorhizobium* (*Alphaproteobacteria*). However, the statistical support for this clustering was low (bootstrap 14%), which did not allow us to conclude about the phylogenetic similarity of the *tls* gene sequences from the above phages and the sequences identified in the phiLM21-LPhs grouped in cluster A (Figure 4b).

Cluster B consisted of two subclusters, B1 and B2 (bootstrap 99%), and included *tls* gene sequences from six phiLM21-LPhs that belonged to different groups based on their nucleotide sequence similarity to phage phiLM21. The cluster also included the *tls* gene sequence of phiLM21 (bootstrap value 99%), indicating that the *tls* sequences identified within phiLM21-LPhs are phylogenetically related to the corresponding gene of phage phiLM21, as determined by cladistic clustering of the terminase sequence (Figure 4b).

Cluster C grouped the *tls* gene sequences found in five phiLM21-LPhs from Groups 1 and 2. Additionally, this cluster included *tls* gene sequences from the phages TM3_3_3 and N28_2, which infect bacteria of the genus *Rhizobium* (bootstrap 100%). The closest branch to this cluster was the branch containing *tls* sequences from two temperate *Sinorhizobium* phages, StopSmel and Aussie. However, the statistical support for this clustering was low (bootstrap 49%), suggesting that the *tls* gene sequences of phiLM21-LPhs within this cluster are more closely related to *tls* sequences of phages infecting *Rhizobium* bacteria than to those infecting *Sinorhizobium*, based on cladistic clustering of the terminase sequence (Figure 4b).

In conclusion, the clustering of phiLM21-LPhs based on nucleotide sequences of the *tls* gene did not correlate with the degree of nucleotide sequence similarity between the prophages and phage phiLM21 (Figure 3, prophage Groups 1–3).

Each of 25 phiLM21-LPhs also contained an ORF, whose product was an integrase according to PHASTEST and NCBI annotation tools (Table S7). Based on primary protein sequence analysis, these integrases were similar to tyrosine integrases. Only the integrase of phiSM11-017 was identical to that of *Sinorhizobium* phage phiLM21 (E = 0, Tables S5 and S7, Figure 3). Interestingly, the integration site for both the indicated prophage and phage was an isocceptor tRNA-Pro(GGG) in the genome of strain SM11 and LM21, respectively. Both sequences of phiSM11-017 and phiLM21 contained a 64 bp fragment identical to the 45 bp sequence of the 3′-end of the tRNA-Pro(GGG) gene in *S. meliloti*, which in phiLM21 was characterized as the *attP* site (coordinates 6 to 69; I = 96.9%). Thus, this sequence could also serve as the *attP* site for phiSM11-017.

The remaining 24 integrase sequences were similar to integrases from three different phages infecting *Alphaproteobacteria* of the families *Hyphomicrobiales*, *Caulobacterales*, and *Rhodospirillales*. For 17 sequences, similarity was shown with the integrase of *Azospirillum* phage Cd (NC_010355). The level of similarity between the primary amino acid sequences of these integrases and that of *Azospirillum* phage Cd varied among the prophages, with integration into the tRNA-Leu(UAA)-031 and tRNA-Lys(CUU)-039 genes (Cov = 79% and 66–74%, respectively; Table S7). In seven phiLM21-LPhs sequences, similarity was observed with the integrase of *Caulobacter* phage Cr30 (NC_025422; E = 4.0 × 10^−10^–1.4 × 10^−8^). Additionally, one of the primary amino acid sequences of the integrase (phiRRI128-016) also showed similarity to the integrase of *Escherichia* phage HK446 (E = 3.6 × 10^−5^; Table S7).

**Figure 5:**
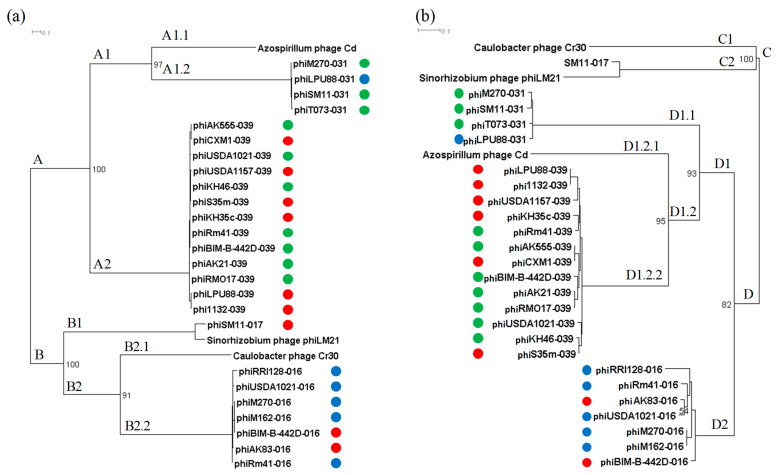
Phylogenetic trees constructed based on (**a**) amino acid sequences and (**b**) nucleotide sequences of phiLM21-LPhs integrases. Colored circles represent groups of phiLM21-LPhs (see Figure 3): blue—Group 1, red—Group 2, green—Group 3. Labels A–D indicate clusters on the phylogenetic trees.

The search and annotation of ORFs corresponding to phage integrases were conducted using amino acid sequences with PHASTEST. To assess the phylogenetic relationship of the integrases with the respective phages, phylogenetic trees were constructed based on both amino acid (aa-*int*) and nucleotide (na-*int*) sequences of phiLM21-LPhs integrases (Figure 5a,b). The tree built on the aa-*int* sequences revealed four subclusters: A1, A2, B1, and B2 (bootstrap 91–100%; Figure 5a). Each included aa-*int* sequences from prophages with varying levels of similarity to phage phiLM21 (Groups 1–3 above; see Figure 5a). The amino acid sequences of integrases similar to those from *Sinorhizobium* phage phiLM21 or *Caulobacter* phage Cr30, as well as the aa-*int* sequences of the corresponding phages, belonged to different subclusters, B1 and B2, indicating their phylogenetic divergence from each other.

Amino acid sequences of phiLM21-LPhs integrases similar to the integrase of *Azospirillum* phage Cd belonged to two subclusters, A1 and A2 (bootstrap 100%). Subcluster A2 was the most numerous and included aa-*int* prophages that were integrated into analogous sequences of isoacceptor tRNA-Lys(CUU)-039. In contrast, subcluster A1 comprised aa-*int* prophages that were integrated into the gene of tRNA-Leu(UAA)-031, as well as aa-*int* sequences of *Azospirillum* phage Cd.

An analysis of the trees constructed based on aa-*int* and na-*int* sequences was performed (Figure 5). It was shown that the phylogenetic trees had different topologies (Figure 5). Nevertheless, clustering of na-*int* sequences according to the prophage integration site was observed both in the amino acid sequence analysis (A1, A2, B1, B2) and in the nucleotide sequence analysis (D1.1, D1.2.2, C2, D2).

However, the similarity of phiLM21-LPhs integrases to *Caulobacter* phage Cr30 and *Azospirillum* phage Cd integrases at the nucleotide level, as revealed by aa-*int* analysis, was not confirmed (Figure 5b). Therefore, it can be concluded that the similarity of aa sequences of integrases does not reflect their phylogenetic relationship but is rather due to the structural features of the proteins necessary for phage binding to the integration site.

Thus, integrase sequences similar to phage integrases at the amino acid level have a very low level of similarity at the nucleotide level with these phages. Consequently, integrase sequences do not reflect the phylogenetic relatedness of sequences associated with a particular phage but can serve as a key sequence for identifying potential phage integration sites.

### 2.3. Analysis of ORFs of phiLM21-LPhs

Among the 25 phiLM21-LPhs, 1872 ORFs were identified, according to PHASTEST. The primary protein products (amino acid sequences, hereafter aa) were grouped into six categories based on their established similarity:

- (aa-i)—proteins of phiLM21 phage;
- (aa-ii)—proteins of other phages, including those infecting bacteria of remote taxa;
- (aa-iii)—proteins of bacteria of the genus *Sinorhizobium*/*Ensifer*;
- (aa-iv)—proteins of phylogenetically remote bacteria;
- (aa-v)—proteins similar to lipocalin family proteins;
- (aa-vi)—hypothetical proteins of unknown origin (Table S8).

Each protein was assigned to a single group based on its PHASTEST annotation. Proteins with similarity to those from known phages or bacteria—including hypothetical, putative, and predicted functional proteins—were categorized into groups aa-i through aa-iv. All products identified as lipocalin family proteins were assigned to group aa-v, regardless of their similarity to viral, bacterial, or plant proteins. Group aa-vi consisted exclusively of hypothetical proteins of unknown origin.

**Figure 6:**
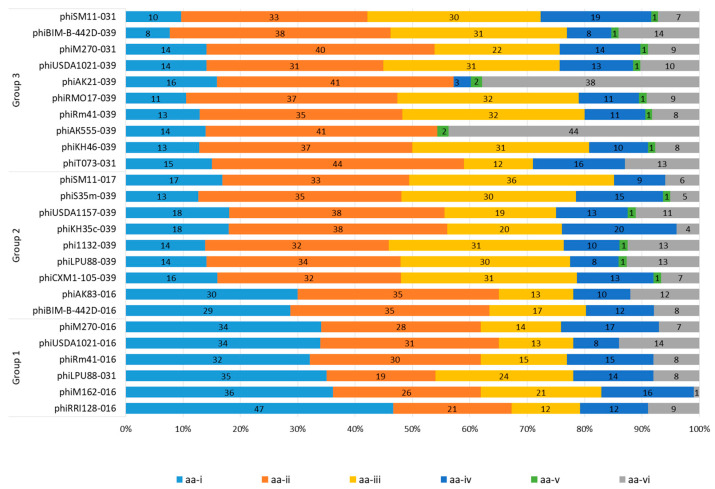
Portion distribution of amino acid sequences encoded by phiLM21-LPhs exhibiting similarity to phages or bacterial genomes. phiLM21-LPhs of distinct groups according to sequence similarity with phage phiLM21 are encoded proteins exhibiting similarity to the following (in %): i—phiLM21; ii—phages infected bacteria of remote taxa; iii—aa sequences of *Sinorhizobium*/*Ensifer* spp.; iv—aa sequences of bacteria of remote taxa; v—lipocalin family aa sequences; vi—aa sequences of unknown origin, according to PHASTEST annotation.

It was established that phiLM21-like prophages, belonging to different Groups (1–3) based on their nucleotide similarity to phage phiLM21, varied in the number of ORF groups aa-i to aa-iv they contained (Figure 6). Prophages of Group 1 contained, on average, 2.3 times more aa-i sequences than prophages of Groups 2 and 3 (36.3%, 18.7%, and 12.7%, respectively). Conversely, for aa-ii and aa-iv, the opposite pattern was observed, with differences of 1.5 times (22.7%, 34.6%, and 37.7% and 16.5%, 25.1%, and 22.1% for Groups 1, 2, and 3, respectively). Statistically significant differences in the occurrence of aa-i and aa-iv sequences were found between Groups 1 and 3 (Χ^2^ = 20.2, *p* = 8 × 10^−3^, α = 0.05), while differences between Groups 1 and 2 were significant at α = 0.1 (Χ^2^ = 9.5, *p* = 0.05).

#### 2.3.1. Groups aa-i and aa-ii

As shown in this work, the highest nucleotide sequence similarity with phage phiLM21 was observed only for phiLM21-LPhs of Group 1. ORFs analysis revealed that their amino acid sequences’ similarity to phiLM21 phage peptides (group aa-i) was, on average, twice as high as that of prophages in Groups 2 or 3. The percentage ratio of ORFs coding for early and late proteins similar to analogous proteins of phiLM21 in the case of prophages of Group 1 was shifted towards products of late-expressed genes (26.2% and 73.8%, respectively). Conversely, similar ORFs in prophages from Groups 2 and 3 occurred in roughly equal proportions (average 0.5/0.5). The difference in the ratio of ORFs for differentially expressed genes between prophages of Group 1 and Groups 2 and 3 was statistically significant (Χ^2^ = 12.3 and 7.9, respectively, *p* < 0.05, df = 1).

An exception was prophages phiBIM-B-442D-016 and phiAK83 (Group 2), which, despite low nucleotide sequence similarity to phage phiLM21 (15% and 14%, respectively), had a similar proportion of aa-i ORFs to that of prophages in Group 1, accounting for 29% and 30% of the total proteins of these prophages (Figure 6).

A total of 383 ORFs coding for aa-i sequences similar to analogous phiLM21 sequences were identified. Functions were predicted for 253 ORFs (66%), related to phage life processes, while 130 ORFs (34%) encoded hypothetical proteins (Table S8).

It was shown that over 95% of prophages contained two ORFs each—one from the early gene group and one from the late gene group (Figure 3). Their products included membrane proteins, transcription antiterminators, tail length tape measure proteins, and proteins involved in head morphogenesis (Table S5). More than 70% of prophages contained ORFs determining the terminase small subunit, while all prophages contained ORFs encoding the terminase large subunit (see above). ORFs predicting tail terminator synthesis and hypothetical proteins were also found in 70% of prophages (Table S5). The remaining ORFs were present in less than 50% of prophages. The consistent conservation of the sequences of these ORFs across prophages from genetically unrelated strains of *Sinorhizobium* from geographically diverse regions suggests that the protein products of these ORFs are crucial for maintaining the properties of intact prophages.

**Figure 7:**
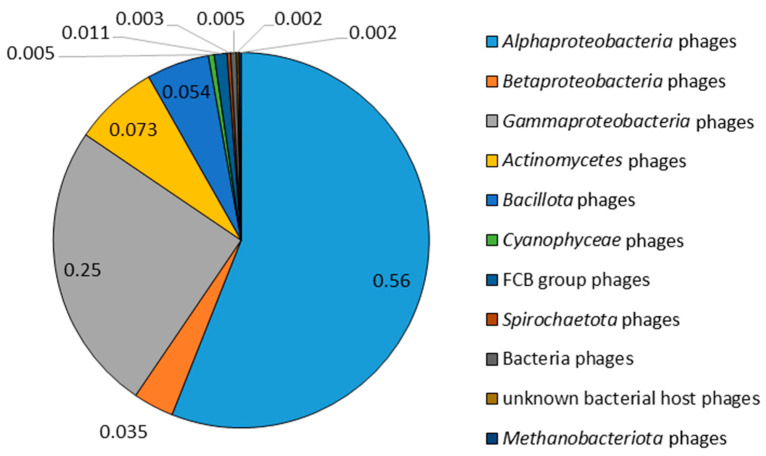
The occurrence of amino acid sequences encoded by phiLM21-LPhs that have similarity to primary protein sequences of phages infecting bacteria of remote taxa.

Within phiLM21-LPhs, 630 ORFs were identified, whose products are similar to primary protein sequences of 108 phages infecting bacteria of remote taxa (aa-ii) (Tables S6B and S8). More than half of these amino acid sequences (56%) were similar to sequences of phages infecting *Alphaproteobacteria* (Figure 7). Sequences similar to phages infecting *Gammaproteobacteria* were twice as rare, comprising 25%. The total share of primary protein sequences similar to those of other phages infecting *Betaproteobacteria*, *Actinomycetes*, *Bacillota*, *Cyanophyceae*, FCB group, *Spirochaetota*, and archaeal *Methanobacteriota* did not exceed 20% (Figure 7, Table S6B).

Among ORFs encoding protein sequences similar to analogous phage proteins, infecting *Alphaproteobacteria* (56%), sequences whose products are similar to proteins of phages infecting bacteria of the genus *Rhizobium* (0.24%) predominated. ORFs similar to those of *Sinorhizobium* phage PBC5 were found with a frequency of 0.07, and those similar to *Sinorhizobium* phages phiM7 and phiM9 were observed only in isolated cases.

For 320 out of 630 amino acid sequences (aa-ii), a function was predicted, whereas the remaining 49% were hypothetical proteins. ORFs with predicted functions mainly resembled proteins involved in processes related to phage DNA. There were no contractile sheath proteins similar to contractile sheath proteins of any other phages detected in phiLM21-LPhs, so it can be assumed that phiLM21-LPhs are characterized by non-contractile tail tubes, as was shown for phiLM21 in [31].

Thus, the analysis of primary protein sequences of phiLM21-LPhs showed their similarity both to proteins from the original phage phiLM21 and to proteins of phages infecting bacteria of remote taxa from other species. Functional prediction indicated that these sequences are necessary for phage viability, but a significant proportion of the protein products are still classified as hypothetical proteins.

#### 2.3.2. Groups aa-iii and iv

On average, each prophage contained from 16 to 41 primary protein sequences (ORFs) that showed similarity to analogous sequences in bacteria. An exception comprised two prophages, phiAK21-039 and phiAK555-039, in which only two ORFs of interest were identified in the first, while in the second, such sequences were entirely absent (Table 1; Figure 6).

A total of 641 out of 1872 ORFs were identified, whose products were similar to amino acid sequences of the 89 bacterial species and the 6 other taxa (*Brucella*, *Rhizobium*, *Pseudomonas*, *Rhizobiales*, *Sinorhizobium*, and *Sinorhizobium*/*Ensifer* group) (Tables S8 and S9A). The majority of ORFs, specifically 416 out of 641 (64.9%), were similar to ORFs from 13 species of the genus *Sinorhizobium* spp. (*Ensifer* spp.) (Tables S8 and S9A1). The most prevalent were ORFs similar to those of *S. medicae* (0.35), whereas the proportion of ORFs similar to *S. meliloti* was nearly four times lower (0.09). Sequences similar to *S. americanum* and to bacteria for which only the genus *Sinorhizobium* was established showed almost equal frequencies (corresponding to 0.16 and 0.14). Rarely, sequences similar to *E. aridi*, *E. glycinis*, *E. psoraleae*, and *E. sesbaniae* were detected, with an average frequency of 0.04. In isolated cases, amino acid sequences similar to those of *E. adhaerens*, *E. mexicanus*, *E. sojae*, *S. arboris*, *S. saheli*, and *S. terangae* were identified (Table S9A1).

Amino acid sequences similar to analogous sequences in bacteria of other taxa were found 1.8 times less frequently (35.1%) (aa-iv; Table S8). In the aa-iv group, sequences characteristic of bacteria of the genus *Rhizobium* (0.30), *Mesorhizobium* (0.11), as well as species *Pseudaminobacter salicylatoxidans* (0.06) and *Agrobacterium tumefaciens* (0.06), predominated. Amino acid sequences similar to those of the remaining 52 species were found in single cases (Table S9A2).

For 53 out of 416 ORFs (frequency 0.13), aa-iii *Sinorhizobium/Ensifer* spp., and for 23 out of 225 ORFs (frequency 0.10), aa-iv, functions were predicted (Table 3, Tables S8 and S9B). Each sequence was assigned to the corresponding COG group, and a Function Code detected by STRING v. 12.0 (searching for similar protein sequences in the database) was also determined and linked to the IMG database. It was shown that the identified amino acid sequences, similar to those in *Sinorhizobium* spp., are primarily involved in metabolic processes (COG groups: Q, P; Table 3). Sequences similar to *Sinorhizobium* spp. and other bacterial species are likely involved in cellular processes and signaling (COG: M, O, V, UW) and in information storage and processing (COG: L). Additionally, a sequence of ammonia monooxygenase, an enzyme involved in ammonia metabolism, was identified within the prophages. Similar sequences were found in *Novosphingobium resinovorum*, which could utilize sulfanilic acid as its sole carbon, nitrogen, and sulfur source.

#### 2.3.3. Group aa-vi (Lipocalin Protein Family)

A lipocalin family protein was identified within 14 prophages (Table 1). Sequences similar to this protein (Cov = 100%, I = 89.9–100%) were present in 11 prophages and showed similarity to a protein identified in *Mesorhizobium* (multispecies protein) (E = 1.8 × 10^−58^–5.8 × 10^−57^). In only one case, the similarity was with a sequence from *Ruania albidiflava* (*Actinobacteria*; E = 2.3 × 10^−18^), according to PHASTEST annotation. Two additional sequences were nearly identical (Cov = 100%, I = 99.4%) and 1.8 times longer than those discussed above; these were annotated as *Temperature-induced lipocalin-1*. The protein sequences identified in prophages phiAK21-039 and phiAK555-039 demonstrated similarity to bacterial membrane proteins (I = 47.0–97.0%, E = 2 × 10^−36^–7.4 × 10^−94^), as well as to plant proteins (I = 35.4–44.5%, E = 1.1 × 10^−26^–9 × 10^−21^). The latter is of particular interest because lipocalin-1 in plants of the genus *Medicago* participates in the development of resistance to cold and oxidative stress [34].

Thus, for the first time, it has been demonstrated that in the prophages phiLM21-LPhs of *S. meliloti*, amino acid sequences corresponding to the lipocalin protein family are present. This is a large group of small extracellular proteins, which are ubiquitously present throughout the tree of life, with the exception of the Archaea domain. Sequences of these proteins are highly dissimilar, while the crystal structures of lipocalins are highly conserved [35]. These proteins are involved in many biological processes, including immune response, pheromone transport, prostaglandin synthesis, retinoid binding, and even interactions with cancer cells [35,36,37].

#### 2.3.4. Group aa-vi

It was shown that 65% of the ORFs identified within phiLM21-LPhs encode hypothetical proteins of viral, bacterial, or unknown origin (Table S8). These represent the so-called “dark matter”—the genetic cargo of prophages whose functions are unknown.

### 2.4. Evidence of Bacterial-Phage “Arms Race”

A search for anti-phage defense system elements in prophage sequences was conducted. Sequences encoding restriction endonucleases or methylases, similar to those found in *Sinorhizobium arboris* (COG: S; prophages phiRMO17-039 and phiBIM B-442D-039; Table 3), were identified. The amino acid sequence showed similarity to the Type I restriction enzyme HsdR of *Serratia odorifera* (I = 45.0%, E = 2.7 × 10^−80^) and may be characterized as a potential participant in defense processes. Sequences potentially involved in cellular defense processes, such as NUDIX hydrolase (prophages phiLPU88-039, phiBIM B-442D-039, and 1132-039), were also identified (Table 3).

Additionally, using the PADLOC web server, individual potential anti-phage system elements were detected, including MTase_II, SspD, and DndC, which were found at an average frequency of 0.04–0.16 (Table 4). Anti-phage system elements were identified in five prophages. ORFs encoding type II methyltransferases (MTase_II) were present in four prophages as a single copy, with two prophages containing two copies each (Table 4). The size of the identified nucleotide sequences ranged from 570 to 2013 bp. A high level of similarity was observed among five copies, with sizes exceeding 1131 bp (I > 97.96%), whereas no similarity was found for the 570 bp sequence (phiAK21-039). ORF encoding the SspD protein was present in two prophages (phiCXM1-105-039 and phiRm41-039) with high similarity (Cov = 100%, I = 99.23%), while no similarity was detected with the corresponding ORFs in prophage phiAK21-039. In prophage phiT073-031, a gene encoding the DndC protein was identified. In bacteria, SspD and DndC proteins are associated with the phosphorothioation system modification module SspABCD [38,39] and DndABCDE [40,41]; however, the affiliation of the identified amino acid sequences to these systems requires further verification.

In addition to the aforementioned potential elements of DNA modification-based systems, sequences encoding components of hypothetical defense systems (Phage Defense Candidates), PDC-S45 and PDC-M32 (phiAK21-039 and phiAUSDA1157-039) were identified.

In the prophage phiRm41-016, using PHASTEST, an ORF was identified whose product showed similarity to CRISPR-associated endoribonuclease Cas2 from *Staphylococcus* phage StB20-like (NC_028821; Cov = 77%, I = 35.62%, E = 3 × 10^−15^), which infects human skin-associated bacteria. This protein also showed similarity (according to blastp) to CRISPR/Cas system-associated endoribonuclease Cas2 identified in many bacteria from remote taxonomic groups, such as the marine bacterium *Alteromonas* sp. MmMcT2-5 and 1_MG-2023 (Cov = 98%, I = 39.1%, E = 5 × 10^−15^), members of the *Bermanella* genus within the *Oceanospirillales* order (Cov = 100%, I = 37.2%, E = 3 × 10^−14^), *Afipia broomeae* (Cov = 98%, I = 36.3%, E = 2 × 10^−13^), the nitrogen-fixing bacterium *Microvirga lupini* (Cov = 100%, I = 47.3%, E = 4 × 10^−17^), as well as hypothetical proteins of symbiotic nitrogen-fixing bacteria *S. meliloti*, *R. leguminosarum*, and *Shinella kummerowiae* (Cov = 99–100%, I = 73.9–100%, E = 2 × 10^−61^ to 4 × 10^−46^).

Thus, integrated intact phage sequences can potentially exert a significant influence on the viability and defensive properties of bacteria. Moreover, they often contain mobile elements. For one-third of the phiLM21-LPhs (28%), sequences encoding transposases were identified. For example, transposases ISRm3 (IS256 family), IS21, and IS5 family transposases were detected, which are present in the genomes of *Sinorhizobium medicae*, *Rhizobium giardinii*, and *Mesorhizobium sophorae*, respectively (Table 3 and Table S9B). The transposase IS1480 was also identified, which was previously found in *Xanthomonas* prophage 33913, as well as ISRSO10-transposase ORFA protein of *Ralstonia solanacearum* GMI1000 prophage (IS66 family transposase; E = 4.7 × 10^−14^). For other transposases, their types could not be determined (Table S6A). These IS elements could destabilize phage-origin integrated sequences, given evidence implicating phiLM21-LPhs in recombination.

### 2.5. Regulators Within phiLM21-LPhs

The switch between lysogenic and lytic life cycles of temperate phages is regulated by a system of regulators, such as in the case of phage λ, where these are the CI and Cro regulators, as well as the anti-termination protein [43]. In the genome of the prophage phiLM21, genes encoding a transcription antiterminator (AWJ26_gp34) and a putative Cro-like protein (AWJ26_gp20) are present; however, the gene encoding the C-repressor protein was not identified in phiLM21. Instead, gene AWJ26_gp19, encoding an XRE family transcriptional regulator and described [31] as a prophage repressor, is present.

A search for ORFs whose products could serve as potential regulators of the life cycle of temperate phages was conducted. It was shown that all phiLM21-like prophages contain ORFs corresponding to a transcription antiterminator of *Sinorhizobium* phage phiLM21 (E = 3.6 × 10^−45^–3.4 × 10^−12^), and none of the prophages contained ORFs similar to the XRE family transcriptional regulator (Table S5). Less than half of the phiLM21-LPhs (44%) contained ORFs whose products were similar to the C-repressor protein of *Rhizobium* phage 16-3 (E = 7.0 × 10^−28^–1.5 × 10^−26^), and half of these prophages (24%) also contained ORFs of putative Cro-like proteins similar to *Sinorhizobium* phage phiLM21 (all ORFs with E = 7.5 × 10^−15^) (Tables S5 and S6B). In addition, ORFs were identified (frequency of 0.4) whose products corresponded to potential repressors of other phages, such as prophage repressors and transcriptional repressors of *Brucella* phage BiPBO1, putative repressors of *Pseudomonas* phage vB_PaeP_Tr60_Ab31 (Table S6A). These ORFs were found in all three groups of prophages, but predominantly in Group 1. Also, in one prophage of Group 1 (phiM270-016) and three prophages of other groups (phiBIM-B-442D-016, phiT073-031, phiAK21-039), no ORF products similar to known phage regulators were identified.

It has been shown that sequences of operators, to which repressors bind, are absent in phiLM21-LPhs. These are identical to those in phage λ (operators oL1-oL3 and oR1-oR3) or *Rhizobium* phage 16-3 (oL1, oL2, oR1, oR2). Based on the obtained data, it can be assumed that either the operator sequences in phiLM21-LPhs differ from those known for phage λ and *Rhizobium* phage 16-3, or the sequences of their operators have undergone changes, the effects of which are unknown.

It can be concluded that some prophages have partially or completely lost their regulatory systems. This loss suggests that their sequences are becoming anchored in the host bacterial genome, and the genes they carry may be expressed during the cell’s life cycle. In contrast, other intact phiLM21-like prophages, which have retained a minimal set of regulators along with lysogenic and lytic cycle elements, could potentially be induced; however, they constitute a small minority (frequency 0.24). Preliminary induction experiments on prophages from this group, using stimuli such as UV light (5.4 J/cm^2^ for 1.5 min; 45 J/cm^2^ for 10 min) or mitomycin-C (0.5 µg/mL for 2 h at 28 °C), did not result in a transition to the lytic cycle. Therefore, it remains unclear whether these prophages can be induced under other conditions. Future studies are planned to investigate this possibility.

## 3. Discussion

Prophages are ubiquitous in bacterial genomes and represent a vast reservoir of genetic information. However, the functional significance of this foreign genetic material remains poorly understood. A substantial amount of data has been accumulated regarding resistance and virulence factors encoded by prophages in pathogenic bacterial strains, as well as their heterogeneity and diversity, and about the genes involved in vital processes of model temperate phages [44,45,46,47,48]. For example, the genes necessary for regulating the transition between the lysogenic and lytic life cycles of temperate phages have been studied using the model phage *E. coli* phage λ [43]. Meanwhile, knowledge about temperate phages in non-pathogenic bacteria, including symbionts of economically important plant species, has been obtained primarily from studies of individual phages [20,27,49].

In this study, a comprehensive comparative analysis was conducted on a group of 25 intact phiLM21-like prophages, which currently represent the most widespread group of prophages in the genomes of *Sinorhizobium meliloti* bacteria, symbionts of plants from the *Fabaceae* family [11]. It was shown that the frequency of occurrence of intact phiLM21-like prophages in the genomes of genetically unrelated *S. meliloti* strains isolated from various remote regions of the world, including an introgressive hybridization center of alfalfa subjected to secondary salinization (the northwestern part of Kazakhstan), was 0.53.

It was demonstrated that nucleotide sequences of phiLM21-LPhs differed slightly in size and GC content both among themselves and from the phiLM21 phage, but significantly differed in the level of coverage of the phiLM21 genome. However, the maximum coverage value did not exceed 34%, and the minimum was 2% (Identity >70%). Only a quarter of the identified intact prophages were potentially phylogenetically related to phiLM21 by cladistic clustering of the terminase sequence, while one-fifth of the prophages were related to rhizobiophages but not to sinorhizobiophages. More than half of the phiLM21-LPhs were related to each other but not to any other known rhizobiophages. The obtained data clearly demonstrate the process of nucleotide sequence diversification within the population of intact prophages present in the genomes of modern *S. meliloti* strains.

Analysis of amino acid sequences encoded by phiLM21-LPhs confirmed the obtained results and showed that ORFs, whose products were similar to phiLM21 proteins, were predominant. At the same time, a significant portion consisted of ORFs whose products were similar to proteins of phages infecting bacteria from phylogenetically remote taxa from *Sinorhizobium* (e.g., *Rhizobium*, *Pseudomonas*, and *Azospirillum*). Additionally, a substantial share comprised ORFs whose products are similar to sequences found in bacteria from taxa that are evolutionarily far apart from *Sinorhizobium* (e.g., *Rhizobium*, *Mesorhizobium*, *Pseudaminobacter salicylatoxidans*, and *Agrobacterium tumefaciens*). This aligns with data obtained for lambda-like phages infecting *Enterobacteriaceae* [50].

Such a significant number of ORFs similar to a broad spectrum of prophages and diverse soil bacteria indicates active recombination processes involving phage and bacterial genomes. Supporting this, the data presented in this article demonstrate the presence of transposase of IS element sequences from different families within phiLM21-LPhs, identified both in temperate phages and in prophages of bacteria from various taxa, which are widely distributed in the genomes of both rhizobia and bacteria from remote taxa.

Analysis of prophage sequences revealed evidence supporting the activity of a phage–microbe “arms race” in the process of evolving defense mechanisms. Various restriction–modification system elements, as well as anti-phage system components of bacteria, were identified within the prophage sequences, which appeared as a result of recombination processes involving phage-derived sequences.

What is the volume of genetic information within prophages that can be characterized as “dark matter”? Our study showed that 1209 ORFs (65% of all ORFs) in phiLM21-LPhs encode hypothetical proteins. Thus, more than half of the prophage ORFs constitute the “dark matter” of the prophage metavirome. Among them, 36.4% of ORFs have phage origin, 46.8% are presumably of bacterial origin, and for 16.9% of ORFs, it is not possible to determine the origin. Intact phiLM21-LPhs, similar to “containers,” harbor genetic information of phage, bacterial, and unknown origins. At the same time, the high prevalence of phiLM21 prophages may indicate that lysogenic strains had ecological advantages, which is consistent with [51].

In this context, questions regarding the possibility of prophages transitioning from the lysogenic to the lytic state, and whether the expression of lysogenic prophage genes in the bacterial host genome is possible, are directly relevant to understanding the functional role of the “dark matter” of phage origin. It is believed that the effect of phage conversion benefits bacterial hosts by providing new functions in a bacterium-phage symbiotic interaction known as lysogenic conversion [52,53,54]. However, all these studies were conducted on pathogenic strains of microorganisms co-evolving with specific phages. The process of prophage gene expression is closely linked to the regulatory systems controlling the life cycles of temperate phages [45]. The expression of prophage genes, including toxin genes, primarily occurs during phage induction, which results in the death of the pathogenic bacterial cell [45]. At the same time, there are data indicating that some phage genes are expressed both in the prophage state and can increase bacterial survival under certain conditions [55]. The impact of temperate phages on non-pathogenic bacteria, which are widely used, for example, in agricultural biotechnology, remains a relatively understudied area.

Analysis of phiLM21-LPhs revealed that only half possess sequences with predicted regulatory functions, although these remain uncharacterized; furthermore, only approximately one-fifth of intact phiLM21-LPhs contain the minimal set of regulators required for the transition from the lysogenic to the lytic cycle, as described in the literature, suggesting that these may be inducible from the bacterial genome. All phiLM21-LPhs were integrated into specific isoacceptor tRNA genes and encoded a tyrosine-type integrase, which recent data suggest may facilitate tRNA-dependent lysogeny [56]. If site-specific integration of the phage into a bacterial tRNA gene leads to its inactivation, phages can compensate for this by encoding a tRNA of the same isotype as the inactivated host tRNA, thereby complementing the loss and ensuring the survival of lysogenic progeny [56]. This mechanism was previously described by our group for prophages similar to *Mesorhizobium* phage vB_MloP_Lo5R7ANS [11]. However, it was not observed in the phiLM21-LPhs studied here, as all host tRNA genes were reconstructed upon phiLM21-LPhs integration, and all phiLM21-LPhs-encoded tRNA genes were different from the host tRNA genes at the integration site. The findings discussed above indicate that phiLM21-LPhs sequences are undergoing “anchoring” within the bacterial genome, a process that, according to [52], is accompanied by co-evolution of the bacterial chromosome and the integrated phage sequence.

The literature contains data indicating that phage-associated genes within genomic islands influence stress tolerance. For example, cyanobacteria of the genus *Synechococcus* express genes in response to oxidative stress conditions [57]. Similarly, genes located in prophages contribute to the virulence program of *Salmonella* [58]. We have shown that genes within genomic islands of *S. meliloti*, which have phage origin but have lost a significant portion of phage genes, including their regulatory elements, can be expressed under normal conditions as well as under osmotic stress conditions [59,60].

The results obtained in our study of prophages in the genomes of non-pathogenic nitrogen-fixing bacterial hosts—which are, in turn, micro-symbionts of leguminous plants—significantly complement and expand the understanding of the fate of site-specific integrated sequences of phage origin within bacterial chromosomes. Simultaneously, the data raise critical questions regarding the evolutionary implications of phage conversion in non-pathogenic bacteria, particularly its role in driving bacterial adaptation and diversification. The accumulated “dark matter” within prophages may further contribute to microbial evolution by serving as a reservoir of genetic variability, potentially facilitating niche specialization or horizontal gene transfer. Collectively, findings from both laboratory experiments and bioinformatics analyses underscore the pressing need for a renewed focus on functional investigations into phage–microbe interactions, which could elucidate their broader evolutionary and ecological consequences.

## 4. Materials and Methods

For analysis, complete genome sequences of 36 *Sinorhizobium meliloti* strains from various regions were used, available in GenBank (RefSeq) (Table 5). The sequences of 33 *S. meliloti* strains have a complete assembly level, while the genomes of AK170, AK555, and CXM1-105 have a contig assembly level. A group of 7 out of the 36 strains was isolated from a region subjected to extreme salinity in northwestern Kazakhstan. The genomes of five of these strains were sequenced, assembled, and annotated at the Laboratory of Genetics and Selection of Microorganisms, FSBSI ARRIAM. The two strains, AK21 and AK83, were analyzed within the framework of international projects INCO-COPERNICUS grant ICA2-CT-2001-10001 and PLADADINFIS EU [61,62] (Table 5).

In this study, genome sequences of 17 sinorhizobiophages listed in Virus–Host DB ([30], accessed 4 April 2025) (Table 6) were used.

The search for prophage sequences in *S. meliloti* genomes was conducted using the web service PHASTEST [73] in deep bacterial sequence annotation mode; the nucleotide genome sequence files (FASTA format) were uploaded. The chromosome sequences of 27 strains were restarted relative to the *oriC* sequence, whereas for 9 strains (S35m, RRI128, MAG283, LPU88, MABNR56, MAG282, LMB1, BIM B-442D, and 1132), sequences were taken directly from GenBank. The boundaries of the identified prophage sequences integrated into tRNA genes were determined according to the Islander algorithm [74], based on the localization of flanking direct repeats.

The bioinformatics classification of phage regions into ‘intact’, ‘questionable’, and ‘incomplete’ was performed using PHASTEST [73]. PHASTEST improves upon earlier versions and enhances annotation of all genomic features, including tRNAs, rRNAs, and protein-coding genes outside prophage regions. The general principles of the program are detailed by Zhou et al. [75]. This classification is based on the size of the phage region, the number of phage-like genes it contains, and its similarity to known phages. It also involves counting the number of identified genes encoding proteins essential for phage structure, DNA regulation, integration, and lysis (so-called ‘cornerstone’ genes), as well as assessing the density of phage-like genes within the region [75]. Nucleotide sequence alignments of genes, phages, and prophages were performed using Nucleotide BLAST (Web BLAST+ 2.17.0: 22 July 2025) (Identity > 70%). Amino acid sequence alignments were performed using Protein BLAST (Web BLAST+ 2.17.0: 22 July 2025). Sequences of phage genes and their protein products were obtained using PHASTEST annotation. tRNA searches within prophage sequences were additionally carried out using tRNAScan-SE v. 2.0 [76].

For phylogenetic analysis, whole-genome data of 90 phages (Table S4) obtained from the NCBI database (submission date July 2025) were used. Comparison of multiple nucleotide sequences of genes was performed using MUSCLE v5 [77]. Phylogenetic trees were constructed based on the sequences of the *tls* gene, encoding the terminase large subunit (TerL), which is used as a phylogenetic marker for phages, and the integrase gene (*int*), using IQ-TREE v.3.0.1 [78] with a maximum likelihood algorithm (1000 bootstrap replicates). The trees were visualized using Dendroscope3 [79].

The search for antiphage defense systems in prophages was performed using a PADLOC web server [42].

Statistical analyses were performed using the PAST software package version 4.03 [80]. The significance of the differences was assessed using the Χ^2^ criterion at α = 0.05.

The following abbreviations were used to describe the results obtained: I—Identity, Cov—Cover, E—E-value.

## Figures and Tables

**Table 1:** Intact *S. meliloti* phiLM21-LPhs.

Strain	phiLM21-LPh ^I^	Integration Site (tRNA Gene)	Length (bp)	GC (%)	Total Number of Protein-Coding Genes	Genes Encoding Proteins Similar To: ^II^						tRNA Gene ^III^
						Phage Origin Proteins		Non-Phage Origin Proteins				
						Total Number	Similar to phiLM21	Hypothetical	*Sinorhizobium*-like (*S. meliloti*)	Other Bacterial Taxa	Lipocalin Family Protein	
1132	phi1132-039	^LYS^_(CUU)_	53,994	59.9	72	33	10	9	22 (2)	7	1	^Met^(CAU)
AK21	phiAK21-039	^LYS^_(CUU)_	52,989	59.2	58	33	9	22	–/–	2	1	–
AK83	phiAK83-016	^SER^_(GCU)_	52,890	59.8	77	50	23	9	10 (4)	8	–	^Val^(CAC)
AK555	phiAK555-039	^LYS^_(CUU)_	50,261	59.8	59	32	8	26	–/–	–/–	1	^fMet^(CAU)
CXM1-105	phiCXM1-105-039	^LYS^_(CUU)_	53,953	59.9	75	36	12	5	23 (0)	10	1	^fMet^(CAU)
KH35c	phiKH35c-039	^LYS^_(CUU)_	52,179	60.4	76	43	14	3	15 (1)	15	–/–	–
KH46	phiKH46-039	^LYS^_(CUU)_	53,258	60	78	39	10	6	24 (1)	8	1	–
M162	phiM162-016	^SER^_(GCU)_	53,422	60.1	70	43	25	1	15 (2)	11	–/–	^fMet^(CAU) ^Met^(CAU)
RMO17	phiRMO17-039	^LYS^_(CUU)_	52,040	59.6	76	36	8	7	24 (2)	8	1	–
RRI128	phiRRI128-016	^SER^_(GCU)_	51,898	60.5	77	52	36	7	9 (1)	9	–/–	^fMet^(CAU)
S35m	phiS35m-039	^LYS^_(CUU)_	53,952	60.3	79	38	10	4	24 (1)	12	1	/
T073	phiT073-031	^LEU^_(UAA)_	55,070	60.3	82	48	12	11	10 (1)	13	–/–	^Met^(CAU)
USDA1157	phiUSDA1157-039	^LYS^_(CUU)_	52,792	60	72	40	13	8	14 (1)	9	1	^Met^(CAU)
BIM B-442D	phiBIM B-442D-016	^SER^_(GCU)_	52,574	60.3	77	49	22	6	13 (2)	9	–/–	–
	phiBIM B-442D-039	^LYS^_(CUU)_	54,659	59.9	78	36	6	11	24 (0)	6	1	^Met^(CAU)
LPU88	phiLPU88-031	^LEU^_(UAA)_	53,460	59.8	74	40	26	6	18 (3)	10	–/–	–
	phiLPU88-039	^LYS^_(CUU)_	52,687	59.9	71	34	10	9	21 (2)	6	1	^Met^(CAU)
M270	phiM270-016	^SER^_(GCU)_	52,173	60.6	76	47	26	5	11 (1)	13	–/–	^Met^(CAU)
	phiM270-031	^LEU^_(UAA)_	55,743	59.3	78	42	11	7	17 (2)	11	1	^Met^(CAU)
Rm41	phiRm41-016	^SER^_(GCU)_	51,921	60.7	74	46	24	6	11 (1)	11	–/–	^Met^(CAU)
	phiRm41-039	^LYS^_(CUU)_	53,565	59.8	85	41	11	7	27 (1)	9	1	^fMet^(CAU)
SM11	phiSM11-017	^PRO^_(GGG)_	50,865	59.5	70	35	12	4	25 (3)	6	–/–	–
	phiSM11-031	^LEU^_(UAA)_	54,164	59.4	83	35	8	6	25 (4)	16	1	^Met^(CAU)
USDA1021	phiUSDA1021-016	^SER^_(GCU)_	52,859	60.8	77	50	26	11	10 (1)	6	–/–	^Met^(CAU)
	phiUSDA1021-039	^LYS^_(CUU)_	53,485	60	78	35	11	8	24 (1)	10	1	–

^I^ Each prophage is named after its host strain and the number of its integration site tRNA gene, according to [11] 016—tRNA-Ser(GCU), 017—tRNA-Pro(GGG), 031—tRNA-Leu(UAA), 039—tRNA-Lys(CUU). Coordinates of phiLM21-LPhs in GenBank accessions are provided in Table S1; ^II^ according to PHASTEST annotation; ^III^ tRNA gene detected in phiLM21-LPhs according to tRNAScan-SE.

**Table 2:** Characteristics of phiLM21-homologous fragments in the phiLM21-LPh sequences (Group 1 prophages).

phiLM21-LPh (Group 1)	Fragments Similar to phiLM21				Cover (%) of phiLM21
	Quantity	Sizes of and to (bp)	Total Length, bp	Average Identity, %	
phiRRI128-016	10	254–6107	17,178	80.7 ± 0.8	33.8
phiM162-016	5	251–7347	13,782	80.7 ± 1.1	27.1
phiLPU88-031	4	1003–7371	13,570	80.8 ± 1.3	26.7
phiRm41-016	6	700–5289	13,309	78.6 ± 1.6	26.2
phiUSDA1021-016	7	302–4947	12,486	79.3 ± 1.3	24.6
phiM270-016	8	325–3742	11,216	79.9 ± 1.0	22.1

**Table 3:** Functional annotation of protein-encoding ORFs in phiLM21-LPhs.

ORF of phiLM21-LPhs					
COG ^I^			Predicted Protein Function	Groups ^II^	
Function Group	Definition (Function Code)	ID			
				aa-iii	aa-iv
Metabolism	secondary metabolites biosynthesis, transport, and catabolism (Q)	COG2931	calcium-binding protein	2	-
		COG2931	protease	1	-
	inorganic ion transport and metabolism (P)	COG5478	low-affinity iron permease family protein	1	-
Cellular Processes and Signaling	cell wall/membrane/envelope biogenesis (M)	COG2244	lipopolysaccharide biosynthesis protein	2	-
	posttranslational modification, protein turnover, chaperones (O)	COG0526	TlpA family protein disulfide reductase	3	3
	defense mechanisms (V)	COG0494	NUDIX hydrolase	3	-
	intracellular trafficking, secretion, and vesicular transport (U); extracellular structures (W)	COG3847	Flp family type Ivb pilin	-	2
Information Storage and Processing	replication, recombination, and repair (L)	COG0593	chromosomal replication initiator protein DnaA	1	-
		COG1793	multifunctional non-homologous end joining protein LigD	-	1
Poorly Characterized	mobilome: prophages, transposons (X)	COG3328	IS256-like element ISRm3 family transposase	1	-
		COG4584	IS21 family transposase	-	1
		COG3666	IS5 family transposase	-	1
	general function prediction only (R)	COG3179	peptidoglycan-binding protein	19	-
	function unknown (S)	COG4748	restriction endonuclease or methylase	2	-
		COG3750	UPF0335 protein NGR_c28390	-	1
-	cell cycle regulation ^III^	-	GcrA cell cycle regulator	5	-
-	signaling ^III^	-	PhnA-like protein	4	-
-	transporter ^III^	-	potassium transporter Kup	3	-
-	oxidative stress and homeostasis ^III^	-	thiol reductase thioredoxin/thioredoxin/redoxin family protein	2	2
-	regulation of cell wall synthesis ^III^	-	SMI1/KNR4 family protein	2	-
-	ammonia metabolism ^III^	-	ammonia monooxygenase	-	4
-	-	-	necrosis-inducing protein	1	-
-	-	-	immunity protein 32	1	-
-	-	-	ATP-binding protein	-	1
-	-	-	FkbM family methyltransferase	-	1
-	-	-	Arc family DNA-binding protein	-	1
-	-	-	EthD family reductase	-	1
-	-	-	three-Cys-motif partner protein TcmP	-	4
Total ORFs				53	23

^I^ Clusters of orthologous groups of proteins, which were determined based on their homology to proteins with predicted functions, as identified by the STRING and IMG databases; ^II^ aa-iii and aa-iv—ORFs whose products are similar to aa of *Sinorhizobium*/*Ensifer* bacteria and bacteria of other taxa, respectively; ^III^ the process is specified according to the predicted protein function.

**Table 4:** Antiphage defense systems elements encoded in prophages (according to PADLOC).

Prophages	Proteins of Antiphage Systems Encoded by Prophag’s Genes *					
	DMS_other			PDC-M32		PDC-S45
	SspD	MTase_II	DndC	PDC-M32B	PDC-M32A	PDC-S45
phiAK21-039	1.8 × 10^−11^/3.7 × 10^−11^	2.6 × 10^−44^/3.3 × 10^−44^	-	-	-	4.3 × 10^−94^/4.7 × 10^−94^
phiCXM1-105-039	3.3 × 10^−15^/1.3 × 10^−14^	2.8 × 10^−80^/1.6 × 10^−70^; 6.6 × 10^−27^/9 × 10^−22^ **	-	-	-	-
phiRm41-039	4.9 × 10^−15^/1.9 × 10^−14^	4.8 × 10^−84^/1.4 × 10^−80^	-	-	-	-
phiT073-031	-	1.8 × 10^−83^/5.4 × 10^−80^; 3 × 10^−26^/2.5 × 10^−21^ **	2.6 × 10^−30^/8.1 × 10^−29^	-	-	-
phiUSDA1157-039	-	-	-	5.9 × 10^−38^/2.4 × 10^−36^	7.5 × 10^−153^/8.3 × 10^−153^	-

*—full sequence E-value/domain E-value (according to PADLOC [42]); **—two copies.

**Table 5:** Genomes of the studied *S. meliloti*.

Strain	NCBI BioSample ID	Strain	NCBI BioSample ID	Strain	NCBI BioSample ID
AK21 ^I^	SAMN08428886	HM006	SAMN07175160	RMO17	SAMN02952139
AK83 ^I^	SAMN00017059	KH35c	SAMN07175161	GR4	SAMN02603224
L6-AK89 ^I^	SAMN22420025 ^II^	KH46	SAMN07175162	CCMM B554 (FSM-MA)	SAMN06284128
AK76 ^I^	SAMN17104055 ^III^	USDA1021	SAMN07175167	Rm41	SAMEA2272434
AK170 ^I^	SAMN10256575 ^IV^	USDA1157	SAMN07175169	T073	SAMN07175166
AK555 ^I^	SAMN08826593 ^IV^	USDA1106	SAMN07175168	M270	SAMN07175164
CXM1-105 ^I^	SAMN08826592 ^IV^	1021	SAMEA3283068	BL225C	SAMN00017103
B399	SAMN06229775	2011	SAMN02603522	RU11/001	SAMEA3146337
B401	SAMN06227501	M162	SAMN07175163	SM11	SAMN02603056
S35m	SAMN16812329	RRI128	SAMN23416898	MAG283	SAMN37646061
LPU88	SAMN37528575	MABNR56	SAMN40039399	MAG282	SAMN37646062
LMB1	SAMN38508088	BIM B-442D	SAMN34164031	1132	SAMN40473144

^I^—strains isolated from region subjected to extreme salinity (see text); ^II^—the strain whose genome was sequenced, assembled, and annotated with the support of WCRC “AgriTechnologies for the Future” (Agreement No. 075-15-2020-920 dated 16.11.2020) [63]; ^III^—the strain whose genome was sequenced, assembled, and annotated under the RSF 20-16-00105 project [64]; ^IV^—strains whose genomes were sequenced, assembled, and annotated under the RSF 17-16-01095 project [65,66,67].

**Table 6:** Phages infecting *Sinorhizobium* spp.

*Caudoviricetes* Virus Lineage	*Sinorhizobium* Infecting Phage	Genome Size (kb)/ CG Content (%)	GenBank Number/ Submission Date	Phage Type	The Enzyme That Determines the Phage’s Integration/Integration Site	Literary Source
Unclassified *Caudoviricetes*	phiLM21	50.8/ 60.6%	NC_029046/2014	temperate	tyrosine integrase/tRNA-Pro(GGG)	[20]
	16-3	60.2/ 59.0%	NC_011103/1998	temperate	tyrosine integrase/tRNA-Pro(CGG)	[27]
	StopSmel	37.8/ 60.8%	OR786374/2023	temperate	transposase/- *	[49]
	Aussie	39.0/ 61.9%	OR786373/2023	temperate	transposase/-	[49]
	PBC5	57.4/ 61.5%	NC_003324/2001	temperate	integrase/-	[20]
	HMSP1-Susan	52.0/ 52.5%	MG214783/2017	- *	integrase **/-	[68]
	NV1.1.1	64.2/ 59.4%	OP484858/2022	- *	-/-	[19]
	phiM6	68.2/ 42.9%	MH700630/2018	- *	transposase/-	[69]
	AP-16-3	61.0/ 59.22%	OP484857/2022	virulent	tyrosine integrase/-	[19]
	AP-J-162	471.5/ 47.1%	PV864765/2025	virulent	-/-	[70]
	phiM5	44.0/ 61.0%	MF074189/2017	virulent	integrase/-	[23]
*Schitoviridae*; *Huelvavirus*	ort11	75.2/ 44.2%	NC_049469/2020	virulent	-/-	[24]
*Pootjesviridae*; *Emnonavirus*	phiM9	149.2/ 49.8%	NC_028676/2015	virulent	-/-	[71]
*Emdodecavirus*	phiM19	188.0/ 49.0%	KR052481/2015	virulent	-/-	[20]
	phiM7	188.4/ 49.0%	NC_041929/2019	virulent	-/-	[20]
	phiM12	194.7/ 49.0%	NC_027204/2013	virulent	-/-	[72]
	phiN3	206.7/ 49.1%	NC_028945/2015	virulent	-/-	[20]

*—no data available; **—the result of this analysis.

## Data Availability

The original contributions presented in this study are included in this article/Supplementary Materials. Further inquiries can be directed to the corresponding author.

## Supplementary Materials

The following supporting information can be downloaded at: https://www.mdpi.com/article/10.3390/ijms26178704/s1.

## Abbreviations

The following abbreviations are used in this manuscript:

phiLM21-LPhs	phiLM21-like prophages
I	Identity
Cov	Cover
E	E-value
*tls*	terminase large subunit
