# Supplementary material for: Dark Matter Carried by Sinorhizobium meliloti phiLM21-like Prophages
Source: Int J Mol Sci. 2025 Sep 6;26(17):8704. doi: 10.3390/ijms26178704 (PMC12429752; doi:10.3390/ijms26178704)
Supplement: Supplementary file 1 [file ijms-26-08704-s001.zip › Tables S1, S3, S4, S7, S8.pdf]

Table S1. GenBank accession numbers and coordinates of phiLM21-LPs.

Strain	GenBank accession numbers	phiLM21-LPh [I]	phiLM21-LPh coordinates
1132	CP148086.1	phi1132-039	610631-664624
AK21	CP026525.1	phiAK21-039	2436956-2383968
AK83	CP002781.1	phiAK83-016	795097-847986
AK555	PZMI02000001.1	phiAK555-039	1859742-1809482
CXM1-105	PZMJ02000001.1	phiCXM1-105-039	715725-661774
KH35c	CP021825.1	phiKH35c-039	1331361-1384539
KH46	CP021822.1	phiKH46-039	2960984-2907727
M162	CP021818.1	phiM162-016	1193347-1246768
RMO17	CP009144.1	phiRMO17-039	2285179-2233140
RRI128	CP088113.1	phiRRI128-016	722447-774344
S35m	CP065020.1	phiS35m-039	2238868-2184916
T073	CP021804.1	phiT073-031	242202-297271
USDA1157	CP021793.1	phiUSDA1157-039	1997056-1944265
BIM B-442D	CP123003.1	phiBIM B-442D-016	1135041-1187614
		phiBIM B-442D-039	2354678-2300020
LPU88	CP135239.1	phiLPU88-031	1867561-1814103
		phiLPU88-039	2416085-2363399
M270	CP021812.1	phiM270-016	2012384-1960212
		phiM270-031	1205888-1261630
Rm41	HE995405.1	phiRm41-016	742162-794082
		phiRm41-039	1887304-1833740
SM11	CP001830.1	phiSM11-017	2402645-2351781
		phiSM11-031	1795437-1849600
USDA1021	CP021800.1	phiUSDA1021-016	1428749-1375891
		phiUSDA1021-039	269820-323304

[I] the name of the prophage includes the name of the strain and the number of the tRNA gene into which this prophage is integrated, according to [11]): 016 – tRNA-Ser(GCU), 017 – tRNA-Pro(GGG), 031 – tRNA-Leu(UAA), 039 – tRNA-Lys(CUU).

Table S3. Genes of phiLM21 covered by phiLM21-LPhs sequences.

**A group of phage genes**	**phiLM21 gene**	**Enzyme / protein of phiLM21**	**phiLM21-LPh of Group-1 ****					
			**phiRRI128-016**	**phiM162-016**	**phiLPU88-031**	**phiRm41-016-**	**phiUSDA1021-016**	**phiM270-016**
Early genes	AWJ26_gp08	exonuclease	+	·	·	·	·	·
	AWJ26_gp09	Erf-like ssDNA annealing protein	+	·	·	·	·	·
	AWJ26_gp10	hypothetical protein	+	·	·	·	·	·
	AWJ26_gp22	hypothetical protein	•	·	·	·	·	+
	AWJ26_gp23	hypothetical protein	+	+	·	+	+	+
	AWJ26_gp24	hypothetical protein	·	·	·	+	+	+
	AWJ26_gp27	DNA methyltransferase	+	·	·	·	·	+
	AWJ26_gp30	hypothetical protein	+	·	·	·	·	·
	AWJ26_gp31	DnaB-like replicative helicase	+	·	·	·	·	·
	AWJ26_gp32	primosomal protein	+	·	·	·	+*	·
Late genes	AWJ26_gp71	terminase small subunit	+	+	+	·	·	·
	AWJ26_gp70	terminase large subunit	+	+	+	·	·	·
	AWJ26_gp69	portal protein	+	+	+	+	+	+
	AWJ26_gp68	hypothetical protein	+	+	+	+	+	+
	AWJ26_gp67	head decoration	+	+	+	+	+	·
	AWJ26_gp66	virion structural protein	+	+	+	+	+	·
	AWJ26_gp65	hypothetical protein	·	+	+	+	+	+
	AWJ26_gp64	head-tail adaptor Ad1	+	+	+	+	+	+
	AWJ26_gp63	head closure Hc1	+	+	+	+	·	+
	AWJ26_gp59	head morphogenesis	+	+	+	+	+	+
	AWJ26_gp58	hypothetical protein	+	+	+	+	+	+
	AWJ26_gp57	tail terminator	·	+	+	+	+	+
	AWJ26_gp56	tail fiber protein	·	+	+	+	+	+
	AWJ26_gp55	hypothetical protein	·	+	+	+	+	+
	AWJ26_gp54	hypothetical protein	·	+	+	+	+	+
	AWJ26_gp52	tail length tape measure protein *	+*	+*	+*	+*	+*	+*

	AWJ26_gp48	tail fiber protein *	·	·	·	+	·	·
	AWJ26_gp44	chitinase	+	+	+	+	+	+
	AWJ26_gp43	hypothetical protein	+	·	·	·	·	·
	AWJ26_gp42	hypothetical protein	+	·	·	·	·	·
	AWJ26_gp41	membrane protein	·	·	·	·	+	·
Total sequences similar to phiLM21 genes:			22	18	17	18	18	17

* truncated gene; · gene is missing; + gene is present; ** Group-1 (Identity > 70%) – see text and Figure 3.

Table S4. Phages infecting *Rhizobium*, *Bradyrhizobium*, and *Mesorhizobium* used in the phylogenetic analysis.

***Rhizobium* phages (NCBI RefSeq)**
**16-3*** (NC_011103.1), AF3 (NC_070887.1), B1VFA (MT770738.1), P11VFA, (MT778840.1), RHEph04 (NC_041908.1), RHEph05 (JX483877.1), RHEph18 (MW980068.1), RHEph19 (MW980069.1), RHph_I1_18 (MN988519.1), RHph_I1_23 (MN988556.1), RHph_I3_11 (MN988510.1), RHph_I4 (MN988552.1), RHph_I9 (MN988520.1), RHph_I36 (MN988508.1), RHph_N1_10 (MN988505.1), RHph_N1_15 (MN988506.1), RHph_N2 (MN988533.1), RHph_N2_6 (MN988543.1), RHph_X94 (MW960029.1), RHph_N3_2 (MN988526.1), RHph_N3_13 (MN988507.1), RHph_N3_19 (MN988527.1), RHph_N28_2 (MN988516.1), RHph_TM3_3_3 (MN988468.1), RHph_TM3_3_14B (MN988496.1), RHph_TM3_14A (MN988472.1), RHph_TM16 (MN988459.1), RHph_TM26 (MN988465.1), RHph_X2_24 (MW960031.1), RHph_X2_25 (MW960032.1), RHph_X2_26 (MW960037.1), RHph_X2_28B (NC_070857.1), RHph_X2_30 (MW980061.1), RHph_X3_9 (MW960034.1), RHph_X66 (MW960030.1), RHph_Y1_10 (MN988487.1), RHph_Y1_11 (MN988558.1), RHph_Y2_4 (MN988546.1), RHph_Y2_7 (MN988514.1), RHph_Y2_11 (MN988547.1), RHph_Y3_1 (MN988490.1), RHph_Y17 (MN988482.1), RHph_Y21 (MN988544.1), RHph_Y25 (MN988538.1), RHph_Y60 (MN988484.1), V1VFA-S (MT778838.1), vB_RglS_P106B (NC_023566.1), vB_RleM_PPF1 (NC_025427.1), RHph_TM30 (MN988521.1), RHph_I20 (MN988539.1), vB_RleA_TRX32-1 (MW023914.1), RHEph01 (NC_047738.1), RHEph08 (NC_047869.1), RHEph03 (JX483875.1), RHph_Y1_20 (MN988488.1), RHEph27 (MW980074.1), RHph_N37 (MN988528.1), RHph_TM33 (MN988492.1), RHph_I38 (MN988529.1), RHEph06 (NC_027296.1), RHph_I1_6 (NC_070852.1), RHEph16 (NC_070856.1), RHEph24 (MW980072.1), RHph_Y38 (NC_070853.1), RHph_N38 (NC_070851.1), RHph_Y2_6 (NC_070854.1), RHph_Y68 (NC_070883.1), RHph_N34 (NC_070884.1), vB_RleM_P10VF (NC_025429.1), P9VFCl (NC_070886.1), RL2RES (NC_070889.1), RL38J1 (NC_070888.1)
***Bradyrhizobium* phages (NCBI RefSeq)**
BDU-MI-1 (KY940711.1), ppBdUSDA122-1 (OP596612.1), ppBeUSDA76-1 (OP596613.1)
***Mesorhizobium* phages (NCBI RefSeq)**
Cp1R7A-A1 (MT188704.1), vB_MloP_Lo5R7ANS (NC_025431.1)

* temperate *Sinorhizobium* phage

Table S7. Integrases identified in phiLM21-LPhs.

<table>
<tr><th rowspan="2">phiLM21-LPhs</th><th rowspan="2">ORF encoding integrase [I]</th><th colspan="3">Primary amino acid integrase sequence</th></tr>
<tr><th>E-value [I]</th><th>Cover (%) / Identity (%) [II]</th><th>Phage containing similar integrase genes</th></tr>
<tr><td>phiAK83-016</td><td>PP_01116</td><td>1.4×10^{-8}</td><td>79 / 27</td><td rowspan="7">Caulobacter phage Cr30</td></tr>
<tr><td>phiM162-016</td><td>PP_01075</td><td>4.6×10^{-9}</td><td>79 / 27</td></tr>
<tr><td>phiBIM B-442D-016</td><td>PP_01066</td><td>5.4×10^{-9}</td><td>79 / 27</td></tr>
<tr><td>phiM270-016</td><td>PP_01062</td><td>4.6×10^{-9}</td><td>79 / 27</td></tr>
<tr><td>phiRm41-016</td><td>PP_01071</td><td>2.8×10^{-9}</td><td>79 / 27</td></tr>
<tr><td>phiUSDA1021-016</td><td>PP_00786</td><td>5.3×10^{-9}</td><td>79 / 27</td></tr>
<tr><td>phiRRI128-016 *</td><td>PP_00687</td><td>4.0×10^{-10} [II]</td><td>79 / 27</td></tr>
<tr><td>phiSM11-017</td><td>PP_02771</td><td>0</td><td>97 / 82</td><td>Sinorhizobium phage phiLM21</td></tr>
<tr><td>phiT073-031</td><td>PP_01805</td><td>1.1×10^{-17}</td><td>79 / 27</td><td rowspan="17">Azospirillum phage Cd</td></tr>
<tr><td>phiLPU88-031</td><td>PP_01814</td><td>5.3×10^{-17}</td><td>79 / 27</td></tr>
<tr><td>phiM270-031</td><td>PP_01909</td><td>5.0×10^{-18}</td><td>79 / 27</td></tr>
<tr><td>phiSM11-031</td><td>PP_02114</td><td>1.6×10^{-17}</td><td>79 / 27</td></tr>
<tr><td>phi1132-039</td><td>PP_00584</td><td>3.7×10^{-17}</td><td>70 / 26</td></tr>
<tr><td>phiAK21-039</td><td>C3L21_12490 [III]</td><td>1.0×10^{-16} [III]</td><td>74 / 26</td></tr>
<tr><td>phiAK555-039</td><td>PP_02162</td><td>1.7×10^{-17}</td><td>71 / 26</td></tr>
<tr><td>phiCXM1-105-039</td><td>PP_02151</td><td>2.3×10^{-17}</td><td>71 / 26</td></tr>
<tr><td>phiKH35c-039</td><td>PP_02149</td><td>1.7×10^{-17}</td><td>71 / 26</td></tr>
<tr><td>phiKH46-039</td><td>PP_02236</td><td>2.1×10^{-17}</td><td>71 / 26</td></tr>
<tr><td>phiRMO17-039</td><td>PP_02188</td><td>1.9×10^{-17}</td><td>71 / 26</td></tr>
<tr><td>phiS35m-039</td><td>PP_02142</td><td>2.1×10^{-17}</td><td>71 / 26</td></tr>
<tr><td>phiUSDA1157-039</td><td>PP_02381</td><td>1.5×10^{-17}</td><td>71 / 26</td></tr>
<tr><td>phiBIM B-442D-039</td><td>PP_02301</td><td>2.9×10^{-17}</td><td>66 / 27</td></tr>
<tr><td>phiLPU88-039</td><td>PP_02374</td><td>3.7×10^{-17}</td><td>70 / 26</td></tr>
<tr><td>phiRm41-039</td><td>PP_02221</td><td>1.7×10^{-17}</td><td>71 / 26</td></tr>
<tr><td>phiUSDA1021-039</td><td>PP_01951</td><td>1.6×10^{-17}</td><td>71 / 26</td></tr>
</table>

[I] according to PHASTEST; [II] NCBI Virus BLAST protein alignment or BLASTp; [III] hypothetical protein C3L21_12490 (according to NCBI annotation tool); * integrase also shows similarities with integrase of *Escherichia* phage HK446 (E-value [I] = 3.6×10^{-5}).

Table S8. Amino acids (aa) sequences encoded by phiLM21-LPhs.

**Group * of aa sequences**		**ORFs**						**Total for two ** groups**	**Frequency per two groups**
		**Function predicted**		**Hypothetical proteins**		**Total**			
		**Number**	**Frequency**	**Number**	**Frequency**	**Number**	**Frequency**		
aa-i	phiLM21 related	253	0.14	130	0.07	383	0.20	1013	0.54
aa-ii	phages	320	0.17	310	0.17	630	0.34		
aa-iii	*Sinorhizobium*/ *Ensifer* spp.	53	0.03	363	0.19	416	0.22	641	0.34
aa-iv	bacteria from other taxa	23	0.01	202	0.11	225	0.12		
aa-v	lipocalin family	14	0.01	0	-	14	0.01	-	-
aa-vi	unknown origin	0	-	204	0.11	204	0.11	-	-
Total		663	0.35	1209	0.65	1872	1.0	-	-

* groups of aa sequences are annotated by PHASTEST (see Text); ** proteins with predicted function and hypothetical proteins.
